# Comparative pharmacokinetics of trimethoprim–sulfadiazine and trimethoprim–sulfamethoxazole in dogs

**DOI:** 10.1186/s12917-026-05604-7

**Published:** 2026-06-04

**Authors:** Carl Ekstrand, Minerva Löwgren, Malin Erkas, Mathias Devreese, Siegrid De Baere, Jules Vo-Thanh, Aude A. Ferran

**Affiliations:** 1 Department of Animal Biosciences, Swedish University of Agricultural Sciences, Uppsala, Sweden; 2 Department of Clinical Sciences, Swedish University of Agricultural Sciences, Uppsala, Sweden; 3 Faculty of Veterinary Medicine, Department of Pathobiology, Pharmacology and Zoological Medicine, Laboratory of Pharmacology and Toxicology, Ghent University, Merelbeke-Melle, Belgium; 4 Université de Toulouse, ENVT, INRAE, INTHERES, Toulouse, France

**Keywords:** Population pharmacokinetics, Protein binding, Nonlinear mixed-effects modelling, Sulphonamide-to-trimethoprim ratio, Free drug concentrations

## Abstract

**Background:**

Potentiated sulphonamides, combining trimethoprim (TMP) with a sulphonamide such as sulfadiazine (SDZ) or sulfamethoxazole (SMX), are widely used in canine medicine. The standard 1:5 dose ratio was initially designed to achieve a plasma concentration ratio of approximately 1:19 in humans, considered optimal for antibacterial synergy. However, species differences in pharmacokinetics may influence whether this ratio is achieved and maintained in dogs. Updated pharmacokinetic data based on modern analytical techniques and unbound (active) drug concentrations are lacking. This study aimed to characterise and compare the pharmacokinetics and plasma protein binding of TMP/SDZ and TMP/SMX in dogs.

**Methods:**

Beagle dogs were administered TMP/SDZ or TMP/SMX intravenously and orally in a crossover design. In addition, TMP was administered orally alone once. Plasma concentrations of TMP, SDZ and SMX were quantified using ultra-high performance liquid chromatography coupled to tandem mass spectrometry (UHPLC–MS/MS). Population pharmacokinetic analysis was performed using nonlinear mixed-effects modelling. Protein binding was determined by ultrafiltration, and free fractions were estimated using linear mixed-effects models.

**Results:**

Oral bioavailability was high for all compounds (93–97%). TMP exhibited substantially higher clearance (0.44 L/h/kg) and a shorter elimination half-life (4.2 h) than SDZ (0.05 L/h/kg; 7.6 h) and SMX (0.026 L/h/kg; 12.6 h). TMP also showed a markedly larger apparent volume of distribution (2.67 L/kg) compared to the sulphonamides (approximately 0.5 L/kg). Protein binding differed between compounds, with population mean free fractions of 0.50 for SDZ, 0.33 for SMX, and 0.43 for TMP. Following both intravenous and oral administration, the free sulphonamide-to-TMP concentration ratios were only close to the proposed 1:19 ratio during the first hours after dosing. Thereafter, the ratios progressively increased due to the more rapid elimination of TMP.

**Conclusions:**

TMP/SDZ and TMP/SMX display distinct pharmacokinetic profiles in dogs, particularly with respect to clearance, half-life and protein binding. The commonly used 1:5 dose ratio does not maintain a stable 1:19 free concentration ratio over a 12 h dosing interval. These findings provide clinically relevant pharmacokinetic data to support rational and species-specific use of potentiated sulphonamides in dogs.

**Supplementary Information:**

The online version contains supplementary material available at 10.1186/s12917-026-05604-7.

## Background

Potentiated sulphonamides (TMPS), combining trimethoprim (TMP) with a sulphonamide such as sulfadiazine (SDZ) or sulfamethoxazole (SMX), have been used in both human and veterinary medicine for decades [1–3]. TMP reduces the minimal inhibitory concentration (MIC) of the sulphonamide component, resulting in mutual potentiation of antibacterial activity [2, 4]. The greatest magnitude of this potentiation has been reported at a TMP: sulphonamide concentration ratio of approximately 1:19 in bacteria causing human infections [2, 5]. In humans, this ratio is achieved in plasma following administration of formulations containing a TMP: sulphonamide dose ratio of 1:5. Because TMP and commonly used sulphonamides such as SDZ and SMX have similar elimination half-lives in humans [6], the deemed optimal 1:19 concentration ratio is maintained throughout the dosing interval. However, recent publications have shown that, in veterinary medicine, different sulphonamides with distinct pharmacokinetic profiles may result in markedly different TMP: sulphonamide concentration ratios in vivo [7, 8]. Earlier pharmacokinetic (PK) studies in dogs also indicate that the elimination half-life of TMP differs from that of SDZ and SMX [9, 10]. Consequently, the optimal plasma concentration ratio may not be achieved and/or sustained over time in this species when using the standard 1:5 dose ratio. In addition, several adverse effects associated with TMPS in dogs have been attributed primarily to the sulphonamide component [11]. Characterising the pharmacokinetics of TMP independently of sulphonamides may therefore be relevant, particularly for understanding drug exposure during prolonged treatment.

The growing global concern about antimicrobial resistance underscores the need for prudent antibiotic use, which is essential to human health. To guide veterinary prescribing, the European Medicines Agency (EMA) has classified TMPS as first-line agents (category D, “Prudent”) for veterinary use [12]. However, to ensure therapeutic efficacy and safeguard animal welfare, the pharmacokinetics and antibacterial effects of these long-established drugs must be thoroughly documented in the target species.

The two PK studies previously conducted in dogs were published more than 30 years ago, at a time when analytical methods were less sensitive and precise than those currently available, *i.e.* liquid chromatography coupled to mass-spectrometry [9, 10]. In addition, those studies did not report individual concentration-time data suitable for *de novo* PK modelling. Neither did they report protein binding, which is essential because it is the free concentration that exerts the antibacterial effect [13]. Therefore, there is a need for updated PK data generated using contemporary quantification techniques and analysed by non-linear mixed-effects modelling (NLME), also referred to as population modelling. An important advantage of population models is their ability to estimate both PK parameters and inter-individual variabilities, the ability to estimate specific sources to variability (covariates) such as age, weight and sex and finally to estimate the residual unexplained variability [14]. The use of NLME is also preferred in small and unbalanced data sets and gives a profound mechanistic understanding of the data compared with other modelling approaches. Furthermore, model outputs can be used to simulate and predict drug exposure under different dosing regimens. Those exposure profiles can then later be integrated with pharmacodynamic models to subsequently predict therapeutic efficacy and to support optimization of dosing regimens. To provide PK data for the support of future development of robust clinical breakpoints and to aid practitioners in decision making, this study aimed to characterise and compare the pharmacokinetics of TMP administered alone and in combination with SDZ or SMX in dogs after intravenous and oral administration, including evaluation of resulting free (active) drug concentration ratios.

## Methods

### Animals

The study was conducted in Beagle dogs from a teaching and research group that were accustomed to handling and sampling. The dog demographics are shown in Table 1. The dogs were housed in groups of two to four dogs per room for social and welfare reasons. The rooms were 20 m^2^ and equipped with raised platforms, toys and resting spaces. During the day, the dogs were let outside in a pen with a doghouse. The dogs had access to a large exercise pen with grass and tunnels once weekly. Dogs were fed a nutritionally complete commercial feed (Royal Canin Gastrointestinal High Fibre) twice daily. Water was available *ad libitum*. Before each drug administration, an intravenous (IV) catheter was placed in the cephalic vein for blood sampling. Before IV administration, an additional IV catheter was placed in the saphenous vein and used for TMPS administration.

**Table 1:** Dog demographics and administered doses for each drug and route of administration

ID	Sex	Age (years)	Weight IV (kg)	Dose IV		Weight PO (kg)	Dose PO	
				mg/kg	mg		mg/kg	mg
trimethoprim/sulfamethoxazole								
1	F	3	14.8	2.0 + 9.7	29 + 144	14.5	11.0 + 54.0	160 + 800
2	F	6	13.6	1.8 + 8.8	24 + 120	13.7	8.8 + 43.8	120 + 600
3	F	3	15	2.0 + 10.0	30 + 152	15	10.7 + 53.3	160 + 800
4	F	6	11.4	1.9 + 9.8	22 + 112	11.4	10.5 + 52.6	120 + 600
5	F	6	13.4	2.0 + 10.0	27 + 136	14.3	11.2 + 55.9	160 + 800
6	F	6	13.8	2.0 + 9.9	27 + 136	14.4	11.1 + 55.6	160 + 800
7	F	6	13	2.0 + 9.8	25 + 128	12.8	9.1 + 46.9	120 + 600
8	F	6	11.8	1.9 + 9.5	22 + 112	11.5	10.4 + 52.2	120 + 600
trimethoprim/sulfadiazine								
9	F	6	13.4	2.7 + 13.4	36 + 180	12.8	4.7 + 23.4	60 + 300
10	F	6	10.5	2.7 + 13.3	28 + 140	10	4.8 + 24.0	48 + 240
11	M	3	19	2.7 + 13.7	52 + 260	18.8	3.6 + 18.1	68 + 340
12	M	3	18	2.9 + 14.4	52 + 260	17.4	3.9 + 19.5	68 + 340
13	F	4	13.8	2.9 + 18.8	40 + 260	13.4	4.5 + 22.3	60 + 300
14	F	4	12.5	3.2 + 16.0	40 + 200	12.5	4.8 + 24.0	60 + 300
15	F	6	14.3	2.5 + 12.6	36 + 180	13.8	3.8 + 18.9	52 + 260
16	M	11	14.8	2.7 + 13.5	40 + 200	14.6	4.7 + 23.3	68 + 340
Trimethoprim								
9	F	7	12.9	-	-	12.9	3.1	40
10	F	7	11	-	-	11	3.6	40
13	F	3	14	-	-	14	2.9	40
14	F	3	13.5	-	-	13.5	3.0	40
15	F	6	13.2	-	-	14.2	2.8	40
16	M	11	14.2	-	-	14.2	2.8	40
17	F	7	10.5	-	-	10.5	3.0	32
18	F	7	11	-	-	11	3.6	40

### Experimental design

A total of 18 dogs were included in the study (Table 1). Eight dogs received TMP/SDZ both IV (3/15 mg/kg) and orally (PO, 4/20 mg/kg), and eight dogs received TMP/SMX both IV (2/10 mg/kg) and PO (10/50 mg/kg), with the order of administration randomised within each treatment group. A wash-out period of at least 3 weeks was applied between treatments. In addition, TMP alone was administered PO (3 mg/kg) to 8 dogs at a later occasion, including 6 dogs previously treated with TMP/SDZ and 2 dogs not previously assigned to any TMPS treatment group. Precise doses for each dog are shown in Table 1. For administration of TMP/SMX, Eusaprim 16 mg/mL + 80 mg/mL (Aspen Nordic, Ballerup, Denmark) was used IV, and Bactrim 80 mg + 400 mg/tablet (Eumedica Pharmaceuticals, Lörrach, Germany) was used PO. For administration of TMP/SDZ, Diatrim 40 mg/mL + 200 mg/mL (Dechra Veterinary Products, Aulendorf, Germany) was used both IV and PO. For PO TMP administration, Idotrim, 160 mg/tablet (Orion Pharma AV, Danderyd, Sweden) was used. The study protocol was evaluated and approved by the Uppsala Animal Experiment Ethics Committee (5.8.18–00776/2023).

Serial blood-samples were collected in EDTA K2 coated tubes at 0, 5, 15, 30 min, and 1, 2, 4, 6, 8, 12, 24, 48, and 72 h following IV administration and at 0, 15, 30, 45 min, and 1, 2, 4, 6, 8, 24, 48, and 72 h after PO administration. The blood samples were centrifuged at 4000 *g* for 10 min, and the plasma was frozen at -80 °C pending analysis.

After IV administration, 100 µL plasma samples were collected at all sampling time points from each of 4 dogs administered each TMPS combination for protein binding determination, resulting in 12 plasma samples per dog. The plasma was transferred to Pall Nanosep Omega 10 K ultrafiltration vials, 10 µL 1 M potassium phosphate buffer (pH 7.5) was added, and then the vials were centrifuged at 4000 g for 40 min at room temperature. The ultrafiltrate was then stored at -80 °C pending analyses.

### Trimethoprim, sulfadiazine and sulfamethoxazole analyses

#### Chemicals and reagents

Analytical standards of SMX, SDZ, TMP and the internal standard TMP-d9 were obtained from Merck Life Science (Hoeilaart, Belgium), whereas internal standards SMX-d4 and SDZ-d4 were purchased from Toronto Research Chemicals. Solvents and reagents used for sample preparation (ethylacetate, acetic acid, dipotassium hydrogenphosphate, potassium dihydrogenphosphate, sodium hydroxide) were of analytical grade (Merck Life Science), and those used for UPLC-MS/MS analysis (acetic acid (AA), acetonitrile (ACN) and methanol) were of ULC/MS quality (Biosolve, Valkenswaard, The Netherlands). Ultrapure water was freshly prepared using a Milli-Q system (Merck Life Science).

#### Determination of total sulphonamide and TMP concentrations in dog plasma

One hundred (100) µL of plasma was transferred to an Eppendorf cup, to which successively 25 µL of an internal standard working solution (SMX-d4, SDZ-d4 at 25 µg/mL and TMP-d9 at 10 µg/mL), 40 µL of a phosphate buffer solution (pH = 6.8) and 100 µL of acetonitrile were added. Following vortex mixing for 1 min and centrifuging (15800 *g*, 10 min, 4 °C), the supernatant was transferred to a 15 mL tube, and 1.5 mL ethyl acetate was added. The sample was extracted by gentle mixing on a roller bank for 15 min, followed by a centrifugation step (1500 *g*, 10 min, 4 °C). The upper part was transferred to a glass tube and evaporated under a gentle nitrogen flow at 40 °C. The dry residue was redissolved in 125 µL of 0.1% (v/v) acetic acid in Milli-Q water and transferred to a conical autosampler vial. A 5-µL aliquot was injected into the ultra-high precision liquid chromatography tandem mass-spectrometry (UPLC-MS/MS) system.

#### Protein binding

Prior to analysis, 25 µL of the thawed filtrate were transferred to an autosampler vial, followed by the addition of 15 µL of the internal standard working solution (SMX-d4/SDZ-d4 at 25 µg/mL and TMP-d9 at 10 µg/mL), 50 µL of mobile phase A (0.1% acetic acid in water) and 25 µL of water/methanol (50/50, v/v). After vortex mixing (15 s), a 5-µL aliquot was injected into the UPLC-MS/MS system.

For each compound, free concentration was modelled as a linear function (linear mixed-effects models) of total concentration constrained through the origin:

1 $$\:{C}_{free}={f}_{u}\cdot\:{C}_{total}$$

where *f*_*u*_ is the free fraction (the fraction unbound to plasma proteins). Temporal changes in *f*_*u*_ were evaluated using linear mixed-effects models with total concentration as a fixed effect and dog as a random effect to account for repeated measures within individuals. All analyses were performed in R (version 4.3.3) using RStudio. Linear mixed-effects models were fitted using the lme4 package. Individual dogs were treated as random effects with random slopes to model the relationship between free and total concentrations. This approach accounts for inter-individual differences in the binding characteristics (the ‘slope’ of the free fraction) across the observation period. Inter-individual variability (IIV) was derived from the variance of these random slopes and expressed as a coefficient of variation (CV%) to quantify the population dispersion, calculated as:

2 $$\:CV\mathrm{\%}=100\cdot\:\frac{\sqrt{{\omega\:}^{2}}}{{f}_{u}}$$

where $\:{\omega\:}^{2}$is the variance of the random slope.

#### Ultra-high precision liquid chromatography tandem mass-spectrometry analysis

Chromatographic separation was achieved on an Acquity UPLC BEH C18 (2.1 × 50 mm, 1.7 μm) column, in combination with a VanGuard precolumn of the same type (2.1 × 5 mm). Mobile phase A was 0.1% (v/v) acetic acid in water, and mobile phase B was acetonitrile. The flow rate was set at 0.45 mL/min, and the following gradient elution program was used: 0 –3.0 min, 90%A/10%B, 3.0–5.5 min, to 5.0% A/95.0% B, 5.5–5.7 min, to 90.0% A/10.0% B, 5.7–7.5 min, 10.0%A/90.0%B. The temperature of the sample compartment and UPLC column was maintained at 8 °C and 30 °C, respectively. The column effluent was sent to a Quattro Premier XE triple quadrupole mass spectrometer (Waters, Antwerp, Belgium). Quantification of the compounds was done by means of component specific MRM (Multiple Reaction Monitoring) transitions: SDZ, mass-to-charge ratio (m/z) 251.2 > 156.1 (quantifier ion) and 251.2 > 92.1 (qualifier ion); SMX, m/z 254.1 > 156.0 (quantifier ion) and 254.1 > 92.2 (qualifier ion); TMP, m/z 291.3 > 230.1 (quantifier ion) and 291.3 > 123.1 (qualifier ion); internal standards: SDZ-d4, m/z 254.9 > 160.0; SMX-d4, m/z 258.0 > 160.2 and TMP-d9, m/z 300.1 > 123.1.

#### Method validation

Method validation of the procedure for the analysis of total (free + bound) and free SDZ/SMX and TMP consisted of analysing spiked blank plasma samples. The matrix-matched calibration curves ranged from 4 to 4000 ng/mL for TMP and from 20 to 100,000 ng/mL for SDZ/SMX. The lower limit of quantification (LLOQ) was 4 ng/mL for TMP and 20 ng/mL for SDZ/SMX.

Accuracy and precision were evaluated using *n* = 6 replicates at the LLOQ and at low, medium, and high concentration levels, prepared in the same way as the calibrator samples. A full validation (within-run and between-run accuracy and precision) of the procedure for the analysis of SMX/SDZ and TMP was performed for chicken plasma (total = free + bound analyte) and for pig plasma (free analyte) (results not shown). Cross-validation (only within-run accuracy and precision) was performed on dog plasma. An overview of the evaluation results for the calibration range, the LLOQ, and the limit of detection is provided in Table S1. The results for accuracy and precision are shown in Table S2.

### Pharmacokinetic analyses

Pharmacokinetic compartment modelling was performed using Monolix 2024R1 (Simulations Plus, Research Triangle Park, North Carolina, U.S.A.) to simultaneously fit NLME models to the experimental TMP, SDZ and SMX concentration-time data. Model evaluation was based on objective function values (OFVs), such as the negative twice the log likelihood (-2LL) and the corrected Bayesian Information Criteria (cBIC), parameter precision and graphical inspection of diagnostic plots (including individual fits, observed vs. predicted concentration, weighted residuals vs. time, and weighted residuals vs. concentration, and the visual predictive check (VPC)).

Both one- and two-compartmental models with a washout period (i.e., drug concentrations were assumed to be zero at the time of drug administration, which was also confirmed < LOQ by the bioanalysis), and first-order elimination were fitted to the TMP, SDZ, and SMX intravenous and PO concentration-time data. For PO administrations, first-order absorption was assumed for the three drugs. The model parameters included: clearance (Cl), volumes of distribution (V), absorption rate constant (ka), and bioavailability (F). For two-compartment models, the peripheral compartment volume (V2) and intercompartmental clearance (Q) were additionally included. Observations below the lower limit of quantification (LOQ) were interval censored, i.e. all the true concentrations were assumed to fall within the interval 0 to LOQ. Except for bioavailability (F), which was considered to follow a logit-normal distribution, all parameters were considered to follow a log-normal distribution. A multiplicative (proportional) residual error model was applied. Inter-individual variability (IIV) was modelled as:

3 $$\:{\theta\:}_{i}={\theta\:}_{tv}\cdot\:\mathrm{e}\mathrm{x}\mathrm{p}\left({\eta\:}_{i}\right)$$

where *θ*_*tv*_ is the typical population value of the parameter, *θ*_*i*_ is the value of the pharmacokinetic parameters in the *i*^th^ dog, and *η*_*i*_ is the deviation from the corresponding population value associated with the *i*^*th*^ dog.

For parameters that were assumed to be lognormally distributed, the estimated standard deviation of the random effects (ω) was converted to a coefficient of variation (CV%) using Eq. 4 and used to describe inter-individual variability (IIV):

4 $$\:CV\%=\sqrt{\mathrm{e}\mathrm{x}\mathrm{p}\left({\omega\:}^{2}\right)-1}\cdot\:100$$

For F, which was considered to follow a logit-normal distribution, CV% was determined using a Monte Carlo simulation. From the distribution of the population parameters, 100,000 samples (x) were drawn, and CV% for F was calculated with the use of Eq. 5:

5 $$\:CV\%=\frac{sd\left(x\right)}{mean\:\left(x\right)}\cdot\:100$$

Shrinkage of the random effects (eta) toward the population mean was calculated as:

6 $$\:shrinkage=1-\frac{SD\left({\eta\:}_{r}\right)}{{\omega\:}^{}}$$

where *SD(η*_*r*_*)* is the empirical standard deviation of the random effects. When the shrinkage for eta exceeded 51%, the individual random effects were considered poorly estimated.

Age, bodyweight and sex were evaluated as covariates. To qualify for inclusion in the final model, a covariate should reduce − 2LL with at least 2 units as well as both IIV and the objective function value (OFV). Additionally, parameter precision should improve, the correlation between the covariate and the parameter should be statistically significant (as assessed by Pearson’s correlation), and the covariate estimate should be significantly different from zero (as determined by Wald’s test). Correlations between parameters within each substance and across different substances were also evaluated.

The secondary pharmacokinetic parameters ( initial and terminal half-lives) were derived from population pharmacokinetic parameter estimates assuming first-order absorption and linear disposition. The absorption half-life was calculated as ln2/kₐ. When one-compartment models were used, the plasma elimination half-life was calculated as ln2·V/Cl. When two-compartment models were used, initial and terminal half-lives were derived from the macro-rate constants (α and β), which were calculated from the corresponding micro-rate constants (k₁₀, k₁₂, k₂₁) obtained from Cl, V₁, Q and V₂.

#### Post hoc bootstrap analyses

A nonparametric bootstrap analysis was performed in Monolix (version 2024R1) to evaluate model robustness and parameter precision. Five hundred bootstrap datasets were generated by resampling individuals with replacement from the original dataset. For each replicate, model parameters were re-estimated using the SAEM algorithm, with the final parameter estimates from the original model used as initial values. Parameter uncertainty was assessed using percentile-based confidence intervals derived from the bootstrap distributions.

## Results

### Pharmacokinetic analysis

**Fig. 1:**
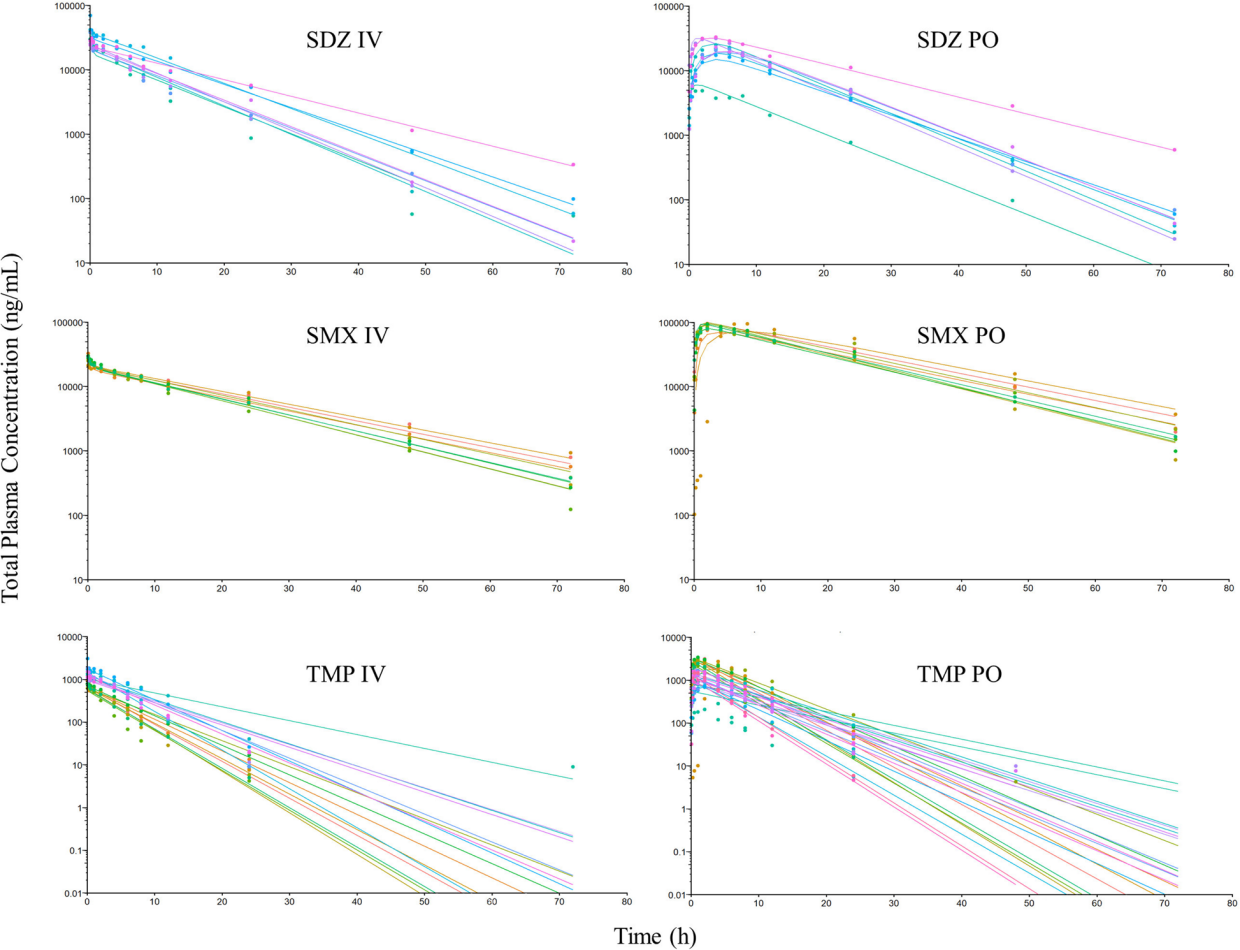
Spaghetti plot showing the observed (symbols) and model-predicted (lines) plasma concentration-time courses after intravenous (IV, left column) and oral (PO, right column) administration of sulfadiazine (SDZ, upper row), sulfamethoxazole (SMX, middle row) and trimethoprim (TMP, lower row) to dogs (*n* = 8 per treatment group). Doses administered were TMP/SDZ IV 3 + 15 mg/kg and PO 4 + 20 mg/kg, TMP/SMX IV 2 + 10 mg/kg and PO 10 + 50 mg/kg, and TMP alone PO 3 mg/kg

**Fig. 2:**
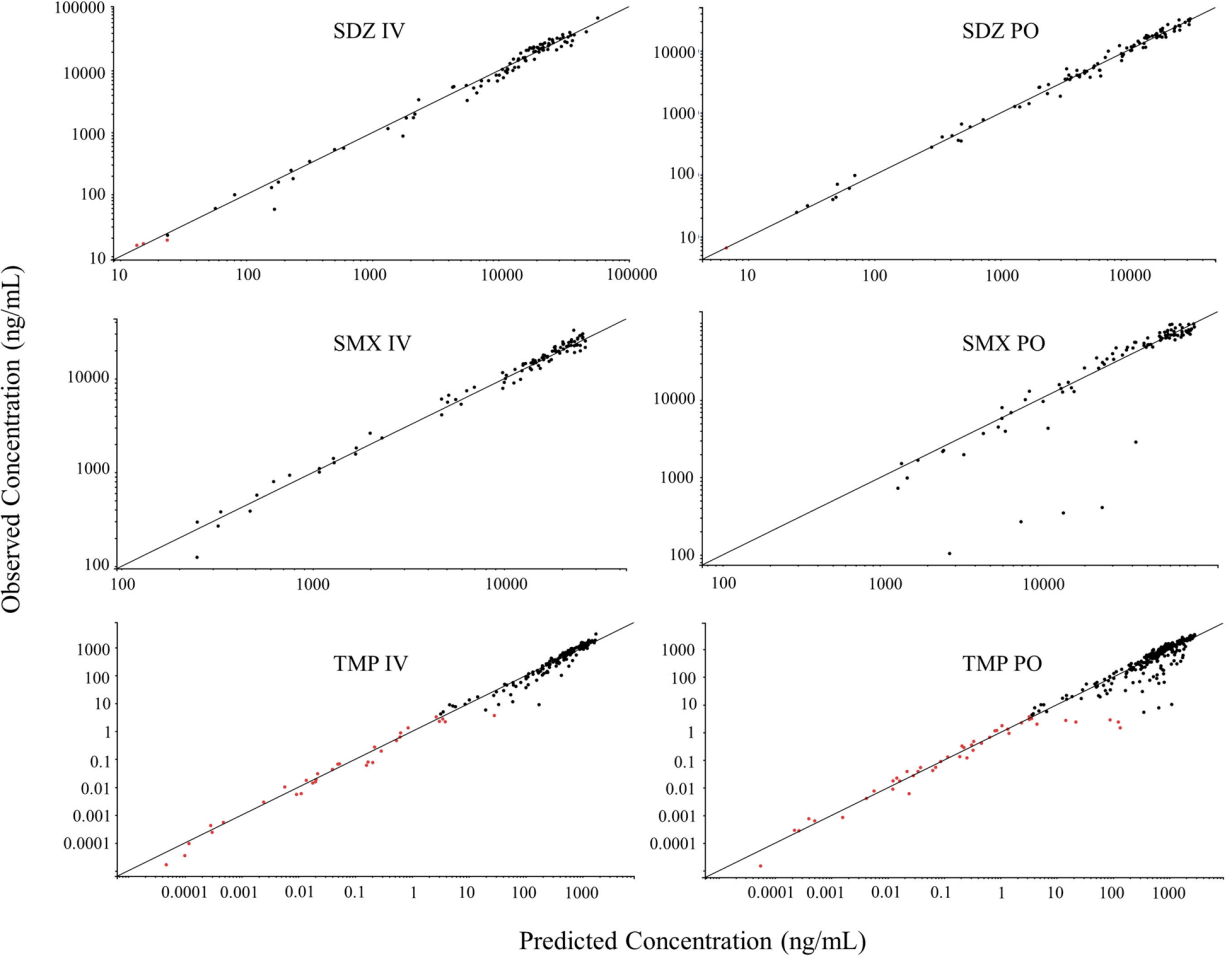
Observed vs. predicted concentrations of sulfadiazine (SDZ, upper row), sulfamethoxazole (SMX, middle row) and trimethoprim (TMP, lower row) after both intravenous (IV, left column) and oral (PO, right column) administration to dogs (*n* = 8 per treatment group). Doses administered were TMP/SDZ IV 3 + 15 mg/kg and PO 4 + 20 mg/kg, TMP/SMX IV 2 + 10 mg/kg and PO 10 + 50 mg/kg, and TMP alone PO 3 mg/kg. The filled black symbols represent quantified concentrations, and the filled red symbols represent observations below the lower limit of quantification. The solid line represents the line of unity (x = y)

**Fig. 3:**
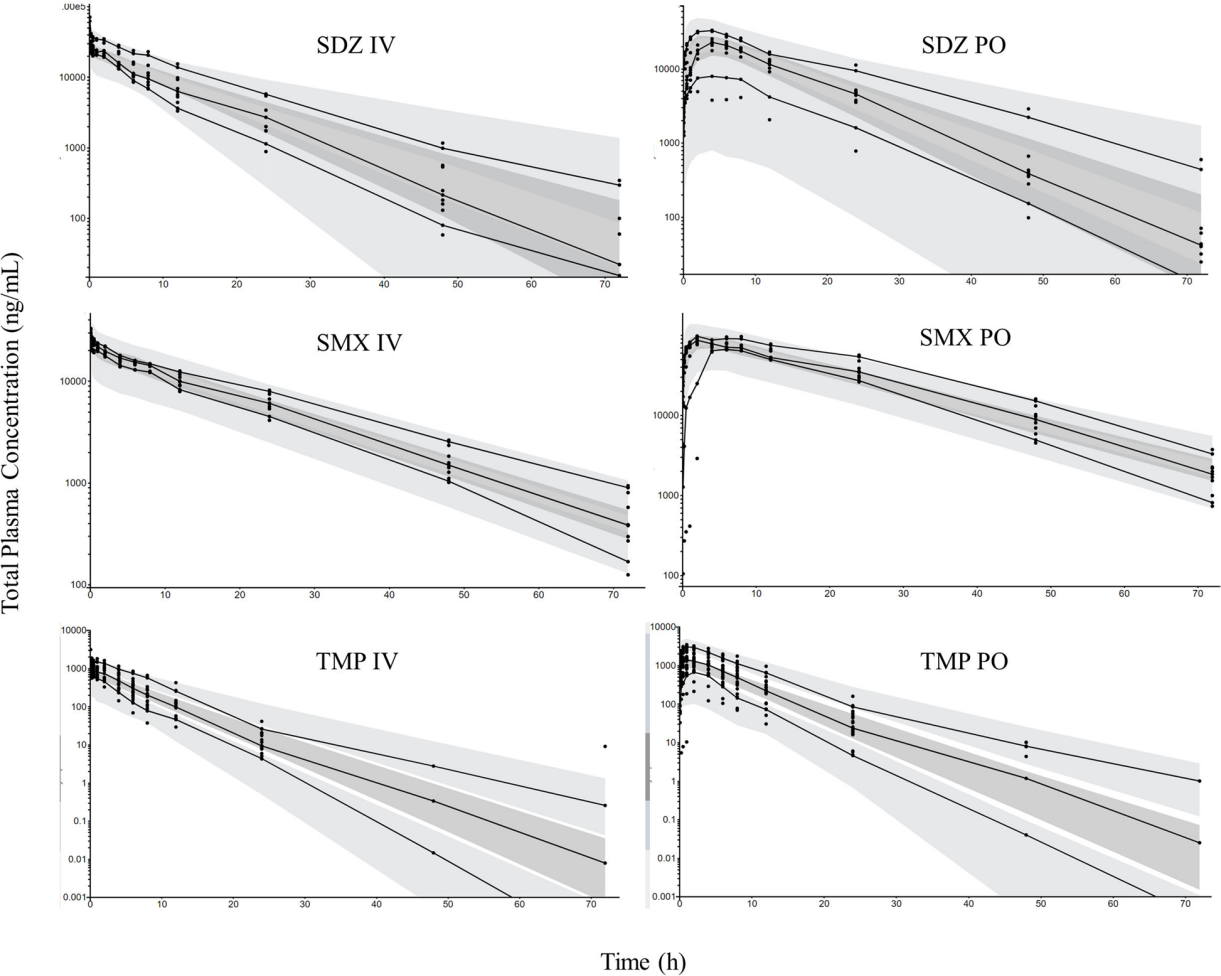
Visual predictive check (VPC) following intravenous (IV, left column) and oral (PO, right column) administration of sulfadiazine (SDZ, upper row), sulfamethoxazole (SMX, middle row) and trimethoprim (TMP, lower row) to dogs (*n* = 8 per treatment group). Doses administered were TMP/SDZ IV 3 + 15 mg/kg and PO 4 + 20 mg/kg, TMP/SMX IV 2 + 10 mg/kg and PO 10 + 50 mg/kg, and TMP alone PO 3 mg/kg. Symbols represent observations, solid lines represent the 10th, 50th (median) and 90th empirical percentiles, respectively. The light grey areas represent the 10th and 90th prediction intervals, and the dark grey area represents the median prediction interval

Overall, the NLME models provided an adequate description of the experimental data of each drug, and each route of administration, with diagnostic plots indicating good predictive performance and model performance appeared acceptable but not entirely free from bias (Figs. 1, 2 and 3). A two-compartment structural model best fit the SDZ and SMX data, whereas a one-compartment structural model best fit the TMP data, as indicated by OFV comparisons and goodness-of-fit plots (PWRES, IWRES, NPDE). No relevant correlation between parameters within each drug and across drugs was identified. No covariates were included in the final model. For the SMX PK parameters F, *V*_*1*_, *V*_*2*_, and Q, and for the SDZ PK parameter Q, shrinkages were > 51%, or the parameter precision was very poor. The SMX parameter Q was fixed to 0.14 L/kg/h based on preliminary results from both IV and PO data in order to improve model stability. No standard deviation of the random effects (and consequently no IIV) was estimated for those parameters. The estimated model parameters are shown in Table 2. The post hoc bootstrap analysis indicated overall model stability, with the main pharmacokinetic parameters being estimated with consistency across resampled datasets. Limited uncertainty was observed for the SDZ PK parameter Q.

**Table 2:** Model-estimated pharmacokinetic parameters for sulfadiazine (SDZ), sulfamethoxazole (SMX), and trimethoprim (TMP) following intravenous and oral administration to dogs (TMP/SDZ: IV 3 + 15 mg/kg, PO 4 + 20 mg/kg, *n* = 8; TMP/SMX: IV 2 + 10 mg/kg, PO 10 + 50 mg/kg, *n* = 8; TMP alone PO 3 mg/kg, *n* = 8)

Parameter	Unit	Typical Value	*R*.S.E. (%)	IIV (%)	Shrinkage (%)	Bootstrap	
						Median	90%PI
Sulfadiazine							
Cl	L/h/kg	0.05	11.1	28.47	23.2	0.05	0.04–0.06
Vc	L/kg	0.27	28.7	33.28	43.3	0.37	0.32–0.49
Vp	L/kg	0.27	18.4	30.54	34.8	0.26	0.14–0.4
Q	L/h/kg	0.91	30.3	-	-	0.93	0.07-3.0
k_a_	1/h	0.65	30.6	74.17	20.9	0.65	0.42–1.07
F	n.a.	0.97	29.8	36.10	36.7	0.98	0.61–0.99
b	n.a	0.2	6.62	-	-	0.2	0.15–0.24
sulfamethoxazole							
Cl	L/h/kg	0.026	4.58	10.76	39.4	0.025	0.023–0.027
Vc	L/kg	0.37	6.02	-	-	0.37	0.32–0.49
Vp	L/kg	0.09	30.0	-	-	0.095	0.0002-0.14
Q	L/h/kg	0.14		-	-		
k_a_	1/h	1.2	28.2	73.83	39.3	1.11	0.65–1.79
F	n.a.	0.91	3.73	-	-	0.92	0.83–0.99
b	n.a.	0.24	5.50	-	-	0.24	0.15–0.36
Trimethoprim							
Cl	L/h/kg	0.43	9.78	40.22	0.57	0.44	0.37–0.51
V	L/kg	2.62	6.38	22.69	14.4	2.62	2.22–3.01
k_a_	1/h	3.69	25.9	118.56	5.3	3.59	2.37–6.02
F	n.a	0.94	8.20	24.32	19.0	0.95	0.76–0.99
b	n.a.	0.41	4.15	-	-	0.4	0.29–0.51

*F* Bioavailability, *k*_a_ Absorption rate constant, *Cl* Clearance, *Vc* Volume of distribution of the central compartment, *V* Volume of distribution, *Q* Intercompartmental clearance, *Vp* Volume of distribution of the peripheral compartment, and b = residual error parameter, *R.S.E.* the relative standard error of the typical value, *IIV * Interindividual variation, *90%PI * 90% percentile interval (5th–95th percentile)

Following IV administration, SDZ displayed a biphasic concentration-time profile, with a rapid distribution phase (initial half-life 0.10 h) followed by a slower terminal elimination phase (terminal half-life 7.5 h). Systemic clearance of SDZ was 0.05 L/h/kg, and the *V* at steady-state was approximately 0.54 L/kg. After oral administration, SDZ absorption was extensive (bioavailability 97%) and moderately rapid (kₐ 0.65 1/h; absorption half-life 1.1 h), resulting in an observed T_max_ typically within 4 h.

Sulfamethoxazole also exhibited biphasic disposition following IV administration and was described by a two-compartment model, with an initial half-life of 0.39 h and a terminal half-life of 12.6 h. Clearance of SMX was lower than that of SDZ (0.026 L/h/kg), and the V at steady-state was approximately 0.46 L/kg. Oral absorption of SMX was rapid and extensive (kₐ 1.13 1/h; absorption half-life 0.61 h; bioavailability 91%), with systemic exposure peaking within the first few hours post-administration.

In contrast, TMP was adequately described by a one-compartment model following IV administration, with markedly higher *Cl* (0.43 L/h/kg) and a substantially larger *V* (2.62 L/kg) than SDZ and SMX. The elimination half-life was approximately 4.2 h. Oral absorption of TMP was rapid (kₐ 3.69 1/h and absorption half-life 0.19 h) and nearly complete (bioavailability 97%), with early systemic appearance and an observed *T*_*max*_ well within 4 h.

### Protein binding and sulphonamide: trimethoprim ratio

**Fig. 4:**
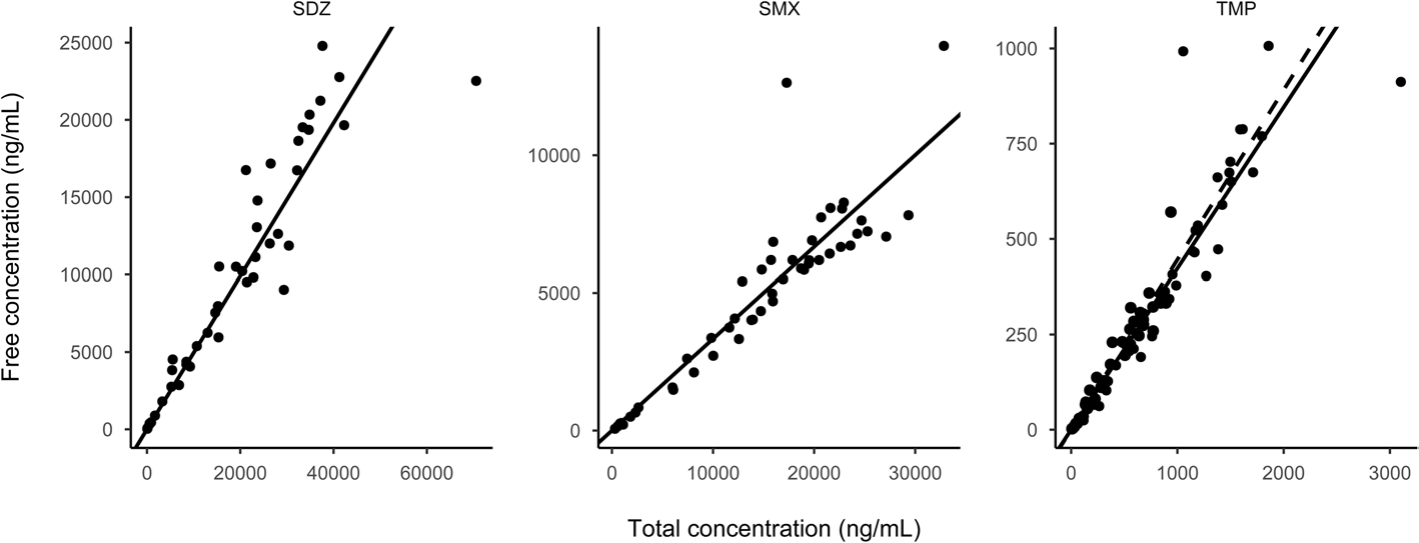
Observed free versus total plasma concentrations of sulfadiazine (SDZ, left panel), sulfamethoxazole (SMX, middle panel), and trimethoprim (TMP, right panel) following intravenous administration of TMP/SDZ (TMP 3 mg/kg + SDZ 15 mg/kg; *n* = 4) or TMP/SMX (TMP 2 mg/kg + SMX 10 mg/kg; *n* = 4) to dogs. Lines represent population free fractions estimated using linear mixed-effects models constrained through the origin; for TMP, separate estimates are shown for each combination (dashed vs. solid lines)

The population mean of the free fraction was 0.50, 0.33, and 0.43 for SDZ, SMX and TMP, respectively (Table 3) based on a study population of four dogs. The Inter-individual variability (expressed as coefficient of variation, CV%) was 7–9%. A proportional relationship between total and free concentrations was observed, suggesting that protein binding was linear for SDZ, SMX and TMP within the studied concentration range (Fig. 4).

**Table 3:** The population mean free fraction (*f*_*u*_) of sulfadiazine (SDZ), sulfamethoxazole (SMX) and trimethoprim (TMP), their 95% confidence interval (95% CI) and interindividual variability (IIV) expressed as the coefficient of variation (CV%)

Compound	f_u_	95% CI	IIV (CV%)
SDZ	0.50	0.45–0.56	8.8
SMX	0.33	0.30–0.37	7.1
TMP	0.43	0.40–0.46	7.3

The median SDZ: TMP free concentration ratio following IV administration ranged from approximately 14 to 22 during the first four hours post-administration (Table 4). Thereafter, the ratio increased gradually, reaching a median of 41 at 12 h and 78 at 24 h post-administration. Following PO administration, the median SDZ: TMP free concentration ratio was 23.9 at 5 min post-administration and remained relatively stable at approximately 23–25 for the first hour. The ratio then increased gradually, reaching median values of 26 at 4 h, 34 at 8 h, 45 at 12 h, and 92 at 24 h post-administration. The median SMX: TMP free concentration ratio following IV administration ranged from approximately 27 to 33 during the first two hours post-administration. Subsequently, the ratio increased progressively to 74 at 8 h, 132 at 12 h, and 515 at 24 h post-administration. Following PO administration, the SMX: TMP free concentration ratio ranged from approximately 20 to 22 during the first 2 h post-administration, after which it increased to median values of 56 at 8 h, 100 at 12 h, and 334 at 24 h.

**Table 4:** Free plasma sulphonamide (sulfadiazine [SDZ] or sulfamethoxazole [SMX]) to trimethoprim (TMP) concentration ratios at different time points following intravenous (IV) and oral (PO) administration. Ratios were calculated from observed total plasma concentrations corrected using the estimated population free fractions (*f*_*u*_)

Time (h)	Median free ratio IV	Range free ratio IV	Median free ration PO	Range free ratio PO
SDZ: TMP				
0.08	23.9	18.3–26.6	21.9	19.0–33.8
0.25	23.6	20.7–26.5	14.8	13.0–23.2
0.5	24.5	20.6–26.2	14.2	11.9–16.8
1	25.4	23.8–26.9	15.3	13.3–18.1
2	26.4	22.9–30.1	16.3	14.1–18.8
4	26.0	19.7–40.6	19.0	15.5–21.5
6	29.0	22.8–51.6	23.1	17.3–31.3
8	33.7	24.6–79.5	29.8	20.9–43.5
12	45.1	14.8–151.1	41.3	29.3–78.1
24	92.4	57.4–678.0	78.3	35.7–228.7
SMX: TMP				
0.08	26.7	24.3–30.4	19.7	4.7–30.4
0.25	26.2	23.9–30.1	20.7	3.9–27.6
0.5	28.5	25.0–31.2	21.7	4.4–26.9
1	30.3	26.9–33.6	21.9	4.6–25.9
2	33.4	29.8–46.2	25.6	6.0–30.2
4	40.5	32.5–93.1	30.4	17.4–42.2
6	52.7	45.6–164.1	38.9	27.9–54.9
8	74.4	60.0–295.1	55.7	41.9–76.4
12	132.4	80.7–311.9	99.9	43.1–118.2
24	514.9	263.1–1694.7	334.4	171.9–1005.2

## Discussion

Potentiated sulphonamides were introduced to veterinary medicine decades ago, and many products registered for or used in animals are composed of one part trimethoprim and 5 parts sulphonamide. This ratio was initially intended to yield a constant plasma concentration ratio of 1:19 in humans, because it was thought to be the optimal ratio for bactericidal effects, a claim that more recently has been challenged. However, as protein binding, absorption, distribution, and elimination may vary substantially between drugs and among animal species, these factors should be explored in a species-specific manner for each substance. Furthermore, the concentration-time profiles may differ according to the route of administration, which can significantly influence antibacterial activity. Consequently, the various routes of administration used in clinical practice warrant systematic investigation. In the present study, dogs were administered TMP/SDZ or TMP/SMX both IV and PO, and TMP PO. The experimental concentration-time data were then analysed using PK modelling, and plasma protein binding was determined.

The final NLME models accurately predicted the observed concentration-time profiles for SDZ, SMX and TMP following both IV and PO administration. Model predictions captured the overall shape and magnitude of the experimental data, and diagnostic plots supported the adequacy of the model. The bootstrap analysis further supported the robustness of the final models, with the main pharmacokinetic parameters being estimated with reasonable consistency across resampled datasets, although the parameter for Q for SDZ was estimated with higher uncertainty. Our results suggest that all three drugs exhibit high oral bioavailability, with estimated bioavailability values close to 100%. The concentration-time profiles also suggested formulation-dependent differences in absorption characteristics. In the final model, a common absorption rate constant (*k*_*a*_) was estimated for TMP across formulations. However, preliminary analyses using formulation-specific ka estimates suggested that TMP tablets exhibited the fastest absorption, TMP/SMX tablets intermediate absorption, and the TMP/SDZ oral solution the slowest absorption. Separate *k*_*a*_ estimates for each formulation were associated with increased parameter uncertainty and reduced model stability, and a common *k*_*a*_ parameter was therefore retained in the final model. Despite these differences in absorption profiles, SDZ and SMX otherwise appeared to exhibit broadly similar pharmacokinetic characteristics in the studied population. The comparatively higher interindividual variability observed for SDZ was likely influenced by an administration-related issue in one individual. For oral administration of TMP/SDZ, a non-labelled injectable formulation was used, and one dog was suspected not to have swallowed the full administered volume, most likely resulting in partial dose loss. This was reflected by lower plasma concentrations of both SDZ and TMP in that individual compared with the other dogs. When TMP was subsequently administered alone in tablet form to the same dog, the resulting exposure was comparable to that of the other animals, suggesting that the lower exposure observed after TMP/SDZ administration was most likely related to incomplete ingestion rather than intrinsic pharmacokinetic variability. Therefore, the choice between TMP/SDZ and TMP/SMX should primarily depend on their intrinsic and comparative antibacterial activity, isolated and in combination with TMP, which would require pharmacodynamic studies of clinically relevant canine pathogens using approaches such as checkerboard assays, time-kill studies, and dynamic in vitro infection models. The present analyses showed that the distribution of TMP was greater than that of SDZ and SMX, and that TMP’s half-life (4.2 h) was much shorter than those of SDZ (7.6 h) and SMX (12.6 h). This is consistent with previous reports [9, 15, 16] and suggests that the rapid decline in TMP concentrations relative to both sulphonamides is primarily driven by markedly higher TMP clearance. This pharmacokinetic asymmetry contributes to the time-dependent divergence in concentration ratios observed over the dosing interval.

The TMP/SDZ and TMP/SMX free plasma concentration ratios were close to the suggested optimal 1:19 ratio only during the first hours after oral administration. Thereafter, the ratios gradually increased, which is consistent with previous studies on TMP/SDZ in dogs [9, 15, 16]. Given the short half-life of TMP, alternative dosing strategies such as additional doses of TMP could potentially later be explored by PK/PD studies. However, the 1:19 ratio most likely reflects an overemphasis on early in vitro observations. rather than a strict in vivo requirement for potentiated antibacterial activity. Indded, both older and more recent studies indicate that synergistic or potentiating interactions between TMP and sulphonamides occur over a range of relative concentrations, rather than at a single fixed ratio of 1:19, and that exposures that deviate from this ratio are also likely to achieve potentiated antibacterial activity in dogs [4, 17, 18]. Such ratio flexibility is likely to be particularly relevant for maintaining antibacterial activity in in vivo conditions, where this study demonstrated that the relative drug concentration ratios change over the dosing interval but also complexifies the PK/PD analysis. A recent study demonstrated that, at any given time, the extent of TMPS synergy depends on the concentrations of both drugs, and that even TMP concentration s up to 22-fold below the MIC can still contribute to synergistic effects [7]. Therefore, conventional PK/PD indices such as T > MIC or AUC/MIC, when applied separately to each drug, are unlikely to accurately predict the efficacy of the combination. This highlights the importance of our study predicting PK profiles of free TMP, SMX and SDZ concentrations after different routes of administration in a dog population. Such data are required to be later integrated with PD data from synergy studies and dynamic in vitro infection models into PK/PD models that will better predict efficacy for different potentiated sulphonamides, different doses and different routes of administration, against canine pathogens such as *Staphylococcus pseudintermedius*.

A crucial methodological difference between the present and previous studies is that ratios in the present study were based on free concentrations. In contrast, earlier studies reported ratios derived from total concentrations. Protein binding was lower than 70% for TMP, SDZ, and SMX. Although these data were obtained from only four animals, very high protein binding in the dog population can likely be excluded. However, greater variability than that reported in this study cannot be ruled out. The use of free concentrations allows the calculation of ratios directly relevant to clinical efficacy, as only the unbound fraction is pharmacologically active and available to exert antimicrobial effects [19, 20]. Because free concentration data for TMP/SDZ and TMP/SMX combinations have not previously been reported, the present study addresses an important knowledge gap and enables PK/PD analyses based on pharmacologically relevant exposure metrics. This experimental study was performed in healthy, middle-aged (3–7 years old) Beagle dogs. The lack of identified covariates and the limited interindividual variability estimated for several PK parameters may partly reflect the relatively homogeneous study population, consisting of healthy Beagle dogs within a narrow age range. Such homogeneity is advantageous for model stability but may underestimate pharmacokinetic variability expected in more heterogeneous clinical populations. Indeed, IIV could not be estimated for some parameters. In addition, other sources of variability exist in a heterogeneous population. Age is one known source of pharmacokinetic variability [21], which has, for example, been demonstrated for tramadol pharmacokinetics in dogs [22]. Although data on the metabolism of TMP, SDZ and SMX in dogs are limited, studies in other species, including humans and rats, suggest that SMX is metabolised by the cytochrome P450 2 C subfamily [23]. In several dog breeds, including the Bearded Collie, Bernese Mountain Dog, Boxer, Briard, French Bulldog, and Irish Wolfhound, a deletion that results in the loss of this gene has been reported [24]. Given that SMX metabolism in other species is closely linked to CYP2C-mediated pathways, breed-dependent differences in CYP2C expression or gene deletion in dogs may not only influence systemic exposure but also alter the relative contribution of metabolic pathways. Such differences are not captured in a homogeneous Beagle population. Moreover, illness can alter PK by altering bioavailability, protein binding, volume of distribution, and clearance [25]. Hence, the pharmacokinetics of potentiated sulphonamides in dogs may be health- and disease-specific, as well as age- and breed-specific, which represents a limitation of the current study and warrants further investigation in clinically ill dogs of different breeds.

## Conclusions

TMP, SDZ, and SMX exhibit distinct pharmacokinetic profiles in dogs, with TMP exhibiting markedly higher clearance and a shorter half-life than the sulphonamides. As a consequence, the free sulphonamide: TMP concentration ratio increased progressively over time following both IV and PO administration, and the commonly used 1:5 dose ratio maintained the historically proposed 19:1 free concentration ratio only during the early post-dose period. By quantifying free plasma concentrations and by modelling PK profilesfor both TMP/SDZ and TMP/SMX combinations after IV and PO administration in a dog population, this study provides mechanistically relevant exposure data that can support future PK/PD analyses and the rational, species-specific optimisation of potentiated sulphonamide therapy in dogs. It could also contribute to the development of canine-specific clinical breakpoints for antimicrobial susceptibility testing by organizations such as the Clinical and Laboratory Standards Institute (CLSI) and the European Committee on Antimicrobial Susceptibility Testing Veterinary Subcommittee (VetCAST). Given the broadly similar pharmacokinetics of SDZ and SMX, selection between these sulphonamides in dogs is likely to depend primarily on their comparative pharmacodynamic activity against relevant pathogens. Practical factors such as formulation characteristics and product labelling may also influence clinical use, particularly for oral administration.

## Supplementary Information

Supplementary Material 1.

## Data Availability

The datasets used and/or analysed during the current study are available from the corresponding author on reasonable request.

## References

[1] Domagk G. Twenty-five years of sulfonamide therapy. Ann N Y Acad Sci. 1957;69(3):380–4. 13488264 10.1111/j.1749-6632.1957.tb49674.x

[2] Bushby SR, Hitchings GH. Trimethoprim, a sulphonamide potentiator. Br J Pharmacol Chemother. 1968;33(1):72–90. 5301731 10.1111/j.1476-5381.1968.tb00475.x PMC1570262

[3] Noall EW, Sewards HF, Waterworth PM. Successful Treatment of a Case of Proteus Septicaemia. Br Med J. 1962;2(5312):1101–2. 20789534 10.1136/bmj.2.5312.1101 PMC1926457

[4] Minato Y, Dawadi S, Kordus SL, Sivanandam A, Aldrich CC, Baughn AD. Mutual potentiation drives synergy between trimethoprim and sulfamethoxazole. Nat Commun. 2018;9(1):1003. 29520101 10.1038/s41467-018-03447-x PMC5843663

[5] Masters PA, O’Bryan TA, Zurlo J, Miller DQ, Joshi N. Trimethoprim-sulfamethoxazole revisited. Arch Intern Med. 2003;163(4):402–10. 12588198 10.1001/archinte.163.4.402

[6] Spicehandler J, Pollock AA, Simberkoff MS, Rahal JJ. Jr. Intravenous pharmacokinetics and in vitro bactericidal activity of trimethoprim-sulfamethoxazole. Rev Infect Dis. 1982;4(2):562–5. 6981172 10.1093/clinids/4.2.562

[7] Boulanger M, Taillandier JF, Henri J, Devreese M, De Baere S, Lacroix M, et al. Population pharmacokinetic modeling of sulfadimethoxine, sulfadiazine and sulfamethoxazole combined to trimethoprim in pigs. Vet Q. 2025;45(1):2565351. 41017374 10.1080/01652176.2025.2565351 PMC12481524

[8] Boulanger M, Taillandier JF, Henri J, Devreese M, De Baere S, Ferran AA, et al. Pharmacokinetic modeling of sulfamethoxazole-trimethoprim and sulfadiazine-trimethoprim combinations in broilers. Poult Sci. 2024;103(11):104200. 39208484 10.1016/j.psj.2024.104200 PMC11399637

[9] Sigel CW, Ling GV, Bushby SR, Woolley JL Jr., DeAngelis D, Eure S. Pharmacokinetics of trimethoprim and sulfadiazine in the dog: urine concentrations after oral administration. Am J Vet Res. 1981;42(6):996–1001. 7283250

[10] Yagi N, Agata I, Kawamura T, Tanaka Y, Sakamoto M, Itoh M, et al. Fundamental pharmacokinetic behavior of sulfadimethoxine, sulfamethoxazole and their biotransformed products in dogs. Chem Pharm Bull (Tokyo). 1981;29(12):3741–7. 7340958 10.1248/cpb.29.3741

[11] Trepanier LA. Idiosyncratic toxicity associated with potentiated sulfonamides in the dog. J Vet Pharmacol Ther. 2004;27(3):129–38. 15189298 10.1111/j.1365-2885.2004.00576.x

[12] Anonymous, Categorisation of antibiotics in the European Union. : European Medicines Agency.; 2020 [Available from: https://www.ema.europa.eu/en/documents/report/categorisation-antibiotics-european-union-answer-request-european-commission-updating-scientific-advice-impact-public-health-and-animal-health-use-antibiotics-animals_en.pdf

[13] Toutain PL, Pelligand L, Lees P, Bousquet-Mélou A, Ferran AA, Turnidge JD. The pharmacokinetic/pharmacodynamic paradigm for antimicrobial drugs in veterinary medicine: Recent advances and critical appraisal. J Vet Pharmacol Ther. 2021;44(2):172–200. 33089523 10.1111/jvp.12917

[14] Mould DR, Upton RN. Basic concepts in population modeling, simulation, and model-based drug development. CPT Pharmacometrics Syst Pharmacol. 2012;1(9):e6. 23835886 10.1038/psp.2012.4 PMC3606044

[15] Frimodt-Möller N, Maigaard S, Madsen PO, Naber KG. Co-trimazine distribution in the canine prostate. Infection. 1979;7(Suppl 4):S345–8. 511347 10.1007/BF01639011

[16] Pohlenz-Zertuche HO, Brown MP, Gronwall R, Kunkle GA, Merritt K. Serum and skin concentrations after multiple-dose oral administration of trimethoprim-sulfadiazine in dogs. Am J Vet Res. 1992;53(7):1273–6. 1497202

[17] Amyes SG. Bactericidal activity of trimethoprim alone and in combination with sulfamethoxazole on susceptible and resistant Escherichia coli K-12. Antimicrob Agents Chemother. 1982;21(2):288–93. 7041815 10.1128/aac.21.2.288 PMC181875

[18] Rosenblatt JE, Stewart PR. Combined activity of sulfamethoxazole, trimethoprim, and polymyxin B against gram-negative bacilli. Antimicrob Agents Chemother. 1974;6(1):84–92. 15828175 10.1128/aac.6.1.84 PMC429051

[19] Craig WA, Ebert SC. Protein binding and its significance in antibacterial therapy. Infect Dis Clin North Am. 1989;3(3):407–14. 2671130

[20] Zeitlinger MA, Derendorf H, Mouton JW, Cars O, Craig WA, Andes D, et al. Protein binding: do we ever learn? Antimicrob Agents Chemother. 2011;55(7):3067–74. 21537013 10.1128/AAC.01433-10 PMC3122431

[21] Ruiz A, DiCristina S. Absorption to Excretion: The Aging Body’s Take on Drugs – A Review of Pharmacokinetic Changes and their Impact on Medication Management. Curr Pharmacol Rep. 2025;11(1):42.

[22] Itami T, Saito Y, Ishizuka T, Tamura J, Umar MA, Inoue H, et al. Comparison of pharmacokinetics of tramadol between young and middle-aged dogs. J Vet Med Sci. 2016;78(6):1031–4. 26875837 10.1292/jvms.15-0638 PMC4937138

[23] Cribb AE, Spielberg SP, Griffin GP. N4-hydroxylation of sulfamethoxazole by cytochrome P450 of the cytochrome P4502C subfamily and reduction of sulfamethoxazole hydroxylamine in human and rat hepatic microsomes. Drug Metab Dispos. 1995;23(3):406–14. 7628308

[24] Karakus E, Prinzinger C, Leiting S, Geyer J. Sequencing of the Canine Cytochrome P450 CYP2C41 Gene and Genotyping of Its Polymorphic Occurrence in 36 Dog Breeds. Front Vet Sci. 2021;8:663175. 33969041 10.3389/fvets.2021.663175 PMC8100205

[25] Morales Castro D, Dresser L, Granton J, Fan E. Pharmacokinetic Alterations Associated with Critical Illness. Clin Pharmacokinet. 2023;62(2):209–20. 36732476 10.1007/s40262-023-01213-x PMC9894673

