# Supplementary material for: Comparative pharmacokinetics of trimethoprim–sulfadiazine and trimethoprim–sulfamethoxazole in dogs
Source: BMC Vet Res. 2026 Jun 4;22:336. doi: 10.1186/s12917-026-05604-7 (PMC13248313; doi:10.1186/s12917-026-05604-7)
Supplement: Supplementary file 1 — Supplementary Material 1. [file 12917_2026_5604_MOESM1_ESM.docx]

**Table S1**. Results of the evaluation of calibration range (correlation coefficient (r), limit of detection (LOD) and lower limit of quantification (LLOQ) for sulfadiazine (SDZ), sulfamethoxazole (SMX) and trimethoprim (TMP) in (filtered) dog plasma. Experiments were performed on 1 day.

| **Analyte** | **Spiked Calibration Range**  **(ng mL^-1^)** | **r^a^** | **LOD^b^**  **(ng mL^-1^)** | **LOQ**  **(ng mL^-1^)** |
| --- | --- | --- | --- | --- |
| ***Unfiltered plasma (total analyte concentration)*** | | | | |
| SDZ | 20 to 100000 | 0.9993 | 0.61 | 20 |
| SMX | 20 to 100000 | 0.9998 | 0.58 | 20 |
| TMP | 4 to 4000 | 0.9986 | 2.79 | 4 |
| ***filtered plasma (free analyte concentration)*** | | | | |
| SDZ | 20 to 100000 | 0.9993 | 1.29 | 20 |
| SMX | 20 to 100000 | 0.9983 | 0.62 | 20 |
| TMP | 4 to 4000 | 0.9979 | 0.26 | 4 |

Note: ^a^r ≥ 0.99; ^b^LOD : calculated concentration that corresponds to a signal-to-noise ratio (S/N) = 3

**Table S2**. Results of the within-run precision and accuracy evaluation for the analysis of sulfadiazine (SDZ), sulfamethoxazole (SMX) and trimethoprim (TMP) in (filtered) dog plasma.

| **Analyte** | **Spiked concentration**  **(ng mL^-1^)** | **Mean concentration ± SD**  **(ng mL^-1^)** | **Precision, RSD**  **(%)** | **Accuracy**  **(%)** |
| --- | --- | --- | --- | --- |
| ***Unfiltered plasma (total analyte concentration)*** | | | | |
| SDZ | 20.0 (n = 6) ^a^ | 21.4 ± 0.5 | 2.4 | 6.9 |
|  | 500.0 (n = 6) | 511.7 ± 16.0 | 3.1 | 2.3 |
|  | 5000.0 (n = 6) | 5196.8 ± 87.5 | 1.7 | 3.9 |
|  | 50000.0 (n = 6) | 46695.1 ± 821.8 | 1.8 | -2.2 |
| SMX | 20.0 (n = 6) | 19.8 ± 1.5 | 7.4 | -0.9 |
|  | 500.0 (n = 6) | 488.5 ± 18.4 | 3.8 | -2.3 |
|  | 5000.0 (n = 6) | 4918.4 ± 86.8 | 1.8 | -1.6 |
|  | 50000.0 (n = 6) | 50756.9 ± 850.6 | 1.7 | 1.5 |
| TMP | 4.0 (n = 6) | 3.7 ± 0.2 | 6.2 | -8.8 |
|  | 100.0 (n = 6) | 95.8 ± 6.9 | 7.1 | -4.2 |
|  | 1000.0 (n = 6) | 936.1 ± 23.5 | 2.5 | -6.4 |
| ***filtered plasma (free analyte concentration)*** | | | | |
| SDZ | 20.0 (n = 6) ^a^ | 19.2 ± 1.7 | 9.0 | -4.3 |
|  | 100.0 (n = 6) | 90.3 ± 5.9 | 6.5 | -9.7 |
|  | 1000.0 (n = 6) | 828.2 ± 66.2 | 8.0 | -17.2 |
|  | 10000.0 (n = 6) | 9158.3 ± 407.3 | 4.4 | -8.4 |
|  | 50000.0 (n = 6) | 46050.7 ± 1575.4 | 3.4 | -7.9 |
| SMX | 20.0 (n = 6) | 17.8 ± 0.8 | 4.7 | -11.2 |
|  | 100.0 (n = 6) | 85.1 ± 5.6 | 6.6 | -14.9 |
|  | 1000.0 (n = 6) | 741.7 ± 94.8 | 12.8 | -25.8 |
|  | 10000.0 (n = 6) | 8040.0 ± 469.2 | 5.8 | -19.6 |
|  | 50000.0 (n = 6) | 41057.5 ± 2408.5 | 5.9 | -17.9 |
| TMP | 4.0 (n = 6) | 3.7 ± 0.5 | 12.8 | -6.6 |
|  | 20.0 (n = 6) | 18.9 ± 1.0 | 5.5 | -5.4 |
|  | 200.0 (n = 6) | 154.1 ± 24.4 | 15.8 | -22.9 |
|  | 2000.0 (n = 6) | 1779.1 ± 110.4 | 6.2 | -11.0 |

Note: ^a^ Within-run accuracy and precision (n ≥ 5); SD: standard deviation; RSD: relative standard deviation; Acceptance criteria for accuracy: LLOQ: ± 20 %, other concentration levels : ± 15 %; Acceptance criteria for precision (RSD_max_): LLOQ: ± 20 %, other concentration levels : ± 15 % [ICH M10].
